# Effectiveness of Strengthening RC Beams Using Composite Materials—An Accelerated Strengthening Method

**DOI:** 10.3390/ma16134847

**Published:** 2023-07-06

**Authors:** Dorota Michałowska-Maziejuk, Barbara Goszczyńska

**Affiliations:** Faculty of Civil Engineering and Architecture, Department of Strength Materials and Building Structures, Kielce University of Technology, al. Tysiąclecia Państwa Polskiego 7, 25-314 Kielce, Poland; bgoszczynska@tu.kielce.pl

**Keywords:** reinforced concrete beams, composite strengthening (CFRP), NSMR method, accelerated strengthening time

## Abstract

The article analyses the results obtained from tests of preloaded reinforced concrete beams strengthened with carbon fibre strips bonded into the concrete reinforcement cover (NSMR method). Adhesive (thixotropic epoxy resin) bonding takes 7 days at 23 °C. The strengthening process was accelerated by heating the strip using a prototype heating device. Tests on the reinforced concrete members confirmed that accelerating the strengthening process is feasible and allowed the selection of the optimal heating temperature to provide the greatest strengthening level. The study primarily aimed to analyse the effectiveness of strengthening applied to the bottom of reinforced concrete beams under sustained loading throughout the adhesive curing process, simulating real conditions on site. Significantly higher strengthening efficiency was achieved with the use of strip heating, which accelerated adhesive cure time and reduced the strengthening execution time from 7 days to 1.5 h. The analysis included the evaluation of the effect of the steel and composite reinforcement ratios on strengthening effectiveness.

## 1. Introduction

In many countries, including Poland, the ground-up construction rate and scale are gradually decreasing relative to renovation and strengthening, which account for more than half of all construction activity. This situation is mainly due to the increased demands on the part of the occupants and users of existing structures or facilities and a need to follow the principles of sustainable development, i.e., taking into account the cost of newly constructed buildings and the environmental impact. This aspect has become particularly important recently when sustainable development, previously understood as “recycling, reduction and reuse”, has been defined as “restoring, renewing and replenishing”. The new approach promotes efforts to maintain, repair and strengthen existing buildings, including those in seismic hazard zones [1,2], rather than demolish and construct new ones. Structural strengthening solutions using composites were first successfully applied in the 1980s [3,4], leading to extensive laboratory studies of composite applications for strengthening concrete structures. The first test of reinforced concrete (RC) beams strengthened with carbon fibre reinforced polymer (CFRP) sheets was performed at the EMPA research institute in Zurich in 1992, using the externally bonded reinforcement (EBR) technique [5]. Further research efforts were targeted at strengthening concrete members of existing buildings or engineering structures, focusing on the efficacy and rate of the procedure. Concurrently, despite the increased material costs, composite strengthening was applied to real systems such as bridges and industrial or public utility buildings [6,7,8,9]. Composites were used for strengthening applications when the cost associated with taking a building or engineering structure out of service for the duration of conventional upgrading vastly exceeded the value of the strengthening made with high-value, lightweight, corrosion-resistant, and easy-to-install fibre-reinforced polymer (FRP) composites [10,11,12,13]. The efficacy of these procedures was a major factor in searching for methods providing improved CFRP strips—concrete adhesion. As a result, prestressed composite materials started to be used [14,15,16,17]. Pioneering research on strengthening RC beams using prestressed CFRP strips was conducted at EMPA in Switzerland [5]. Another technique for improving adhesion and hence the effectiveness of strengthening RC members was the application of passive gluing of composite reinforcement into the concrete cover—the NSMR method [18,19,20,21,22]. The need to expand and add to the knowledge of this method has attracted research globally. 

The NSMR method consists of bonding composite laminates in the pre-cut grooves in the concrete cover filled with an adhesive. Compared with the externally bonded reinforcement, this system provides improved adhesion and protects the glued composite from mechanical and fire-related damage. The studies found in the literature mainly report on mounting the strips or bars on members in an inverted position, which improves quality and facilitates execution but fails to reflect the real on-site conditions [23,24,25,26,27]. Adhesive cure time is a vital issue in strengthening with composite materials. The addition of heat to reduce the curing time was reported in many studies [16,28] in which the application of composite strips to the external surface of components was tested. 

The primary objective of this paper was to analyse the efficacy of strengthening RC beams with CFRP composite materials using the near-surface-mounted reinforcement (NSMR) method. The strips were mounted on the soffit of members subjected to loading to simulate the actual strengthening conditions in engineering practice. The results of testing ten RC beams under monotonic loading to failure were the basis for the analysis. Two percentages of steel bar reinforcement, two percentages of composite reinforcement, and thermally accelerated adhesive curing in the NSMR method were applied. The reduction in the time required by the strengthening procedure was achieved using a prototype Sika^®^CarboHeater II unit. The most effective temperature for heating the adhesive was determined in preliminary tests. The main achievement of this research was reducing the adhesive curing time from 7 days to 1.5 h, thus reducing the strengthening execution time. Another effect achieved in this study was the higher efficiency of strengthening RC beams using “composite strip heating” compared to beams strengthened without heating. 

## 2. Materials and Method 1

### 2.1. Evaluation of the Possibility of Accelerating Strengthening Time Using CFRP Strips in the NSMR Method

#### 2.1.1. Accelerated Strengthening Procedure—Components Used

**Figure 1:**
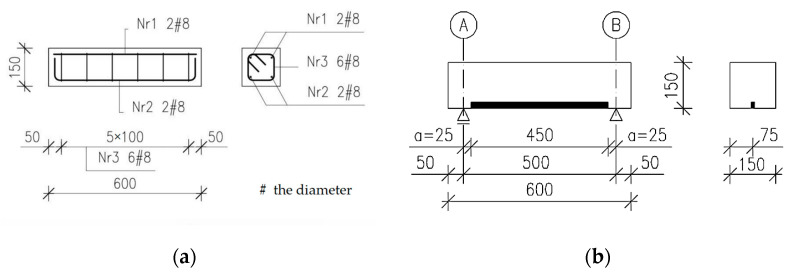
Details of tested elements–steel reinforcement (**a**) [32]; Method of strengthening of elements (**b**).

To evaluate the possibility of accelerating the NSMR strengthening process, 15 RC elements were cast with dimensions of 0.15 × 0.15 × 0.60 m. The elements were reinforced with four 8 mm bars made of ribbed steel of class A-IIIN, grade BST500S with two bars at the top and two bars at the bottom (see Figure 1a). The stirrups were made of the same class and grade of steel with 6 mm diameter bars spaced at 100 mm. For strengthening with the NSMR method, 20 mm high spacers were used during concreting. 

NSMR strengthening consists of cutting out a groove in the concrete cover of an element, filling it with adhesive and then embedding a carbon fibre strip in the so-prepared groove. This study used Sika^®^CarboDur^®^ S NSM 1.030 system to strengthen the RC elements. The strip was bonded into the concrete cover using SikaDur^®^-330, a two-component (A + B) solvent-free, thixotropic epoxy resin. The details of the laminate and adhesive are available in the product information sheets [29,30]. The time required to strengthen an element is closely related to the adhesive’s cure time, which depends on its chemical composition. Since the B-component of the adhesive used is an organic chemicals-based hardener containing an amine group, the increase in the temperature of the strip above room temperature causes “hot” curing of the adhesive [31]. The strengthening of the elements was performed in an inverted position. Details of tested elements (Figure 1a) and the method of strengthening are shown in Figure 1b.

**Figure 2:**
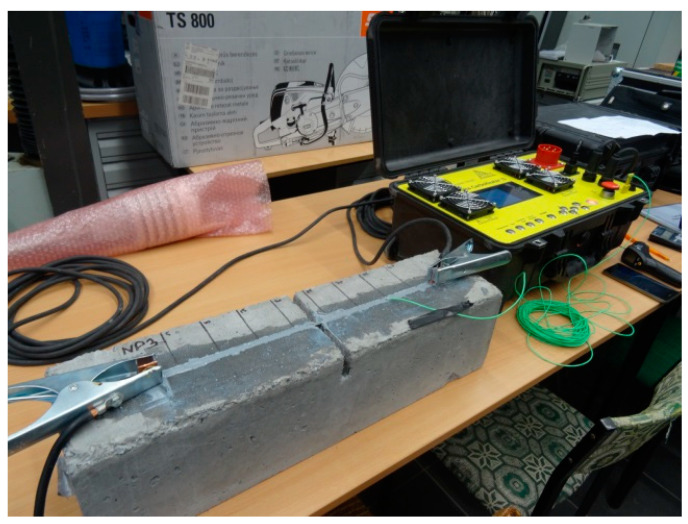
View of reinforced concrete element with attached prototype heating device.

**Figure 3:**
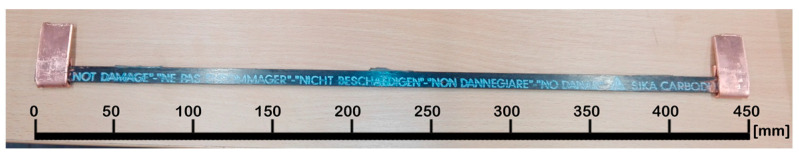
View of CFRP strip with copper flats.

Sika^®^CarboHeater II (Figure 2) was used to heat the composite (CFRP strip) [33]. The operation of the device relies on the flow of the current through carbon fibre strips. Since carbon fibres are good conductors, the current flow heats the strips and adhesive surrounding the strips, thereby shortening the entire strengthening process. The wires were tied with special wire clips. The copper flat clips were clamped on the strip ends before gluing (see Figure 3) and extended past component ends (see Figure 2). Using the prototype heating device reduced the adhesive cure time from 7 days (Product Information Sheet [29]) to 1.5 h. Three test temperatures, t_1_ = 60 °C, t_2_ = 70 °C, and t_3_ = 80 °C, were used to select the optimal heating temperature for the CFRP system. The heating temperature was measured using a thermocouple wire embedded in the adhesive at the hottest point—at mid-length of the heated carbon strip. 

#### 2.1.2. Testing

A total of fifteen RC elements were divided into five three-member series. Series 1 consisted of non-strengthened elements, while each subsequent series included elements strengthened using the Sika^®^CarboDur^®^ S NSM 1.030 CFRP systems varied by adhesive cure time and temperature. Based on Sika’s experience in the application of Sika^®^CarboDur^®^ systems [28,34,35], an equal strip heating duration was assumed for the components using the adhesive with accelerated cure time. For Series 2, 3 and 4, the adhesive cure process (strip heating duration) took 1.5 h at three different heating temperatures. In Series 5, the cure time was 7 days at an ambient temperature of 23 °C [29]. Series 5 elements were loaded seven days after strengthening, while series 2, 3 and 4 elements, strengthened in an inverted position using the prototype heating device, were loaded immediately after strengthening. For flexural failure, all specimens to be strengthened were incised at mid-span in the tension zone of the section and the tension reinforcement was cut. Information about the elements tested is compiled in Table 1.

**Figure 4:**
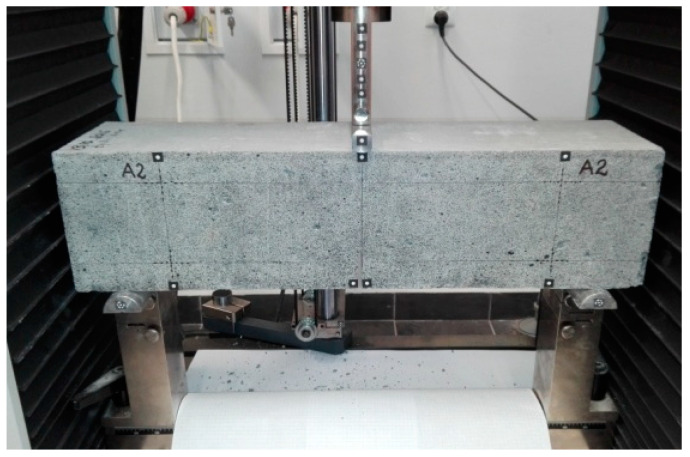
View of the test stand with the test element.

The RC elements were tested in a three-point bending scheme. Due to the capabilities of the test stand, the span of the element in the axes of the supports was equal to l_eff_ = 500 mm. The elements were subjected to a monotonic load with the concentrated force F increasing at a rate of 0.4 kN/min until the specimen failed. The concentrated force was applied at half the distance from the axis of both supports, i.e., at ½ the span (leff.) A view of the test stand is shown in Figure 4. 

#### 2.1.3. Result 1

**Figure 5:**
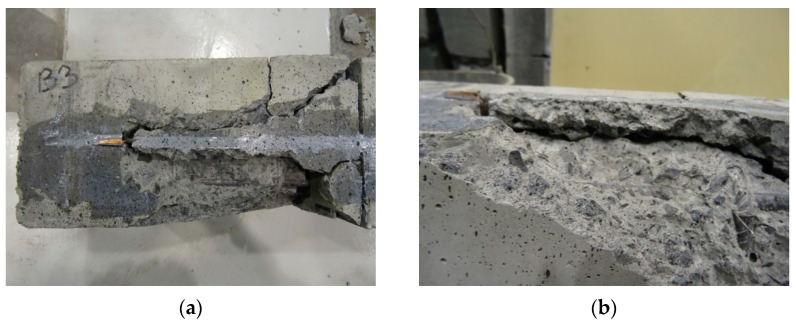
RC element failure: (**a**) underside of series 2 element; (**b**) partial sliding out of the strip from the copper flat bar clip, together with its debonding from the concrete cover (underside of the reinforced element).

Debonding was the primary failure mode in all elements under test. The CFRP strip detached with a portion of the concrete cover. The failure was rapid, regardless of whether the adhesive curing process was accelerated or proceeded according to the product information sheet [29]. In the elements of series 2, 3 and 4, the carbon fibre strip detached together with the concrete cover in the shear zone, and the detachment length almost reached the point of load application. In addition, in series 2 the strip slid out of the copper flat bar clip acting as a conductor (see Figure 5b). The strips in series 5 elements debonded at a greater distance from the shear zone, in a small section closer to the centre of the span. The concrete cover separated from the strip. As demonstrated, the accelerated adhesive bonding process did not result in a different failure mode than bonding under normal temperature conditions. It was observed that the debonding length decreased with the increasing strip heating temperature. The smallest length was observed in the case of the element strengthened with the adhesive cure time of seven days. Failure of the RC element is shown in Figure 5.

The recorded destructive forces of RC elements were used to determine the bending moment at which they failed. The strengthening efficiency of the tested elements was determined based on the strengthening level *η_u_*_,_ relative to non-strengthened elements, from the following formula:

(1) $$\eta_{u}=\frac{M_{U}-M_{U0}}{M_{U0}}$$

where *M_U_*—the failure moment of the strengthened element, *M_U0_*—the failure moment of the non-strengthened element.

The values of the determined strengthening level are shown in Table 2.

**Figure 6:**
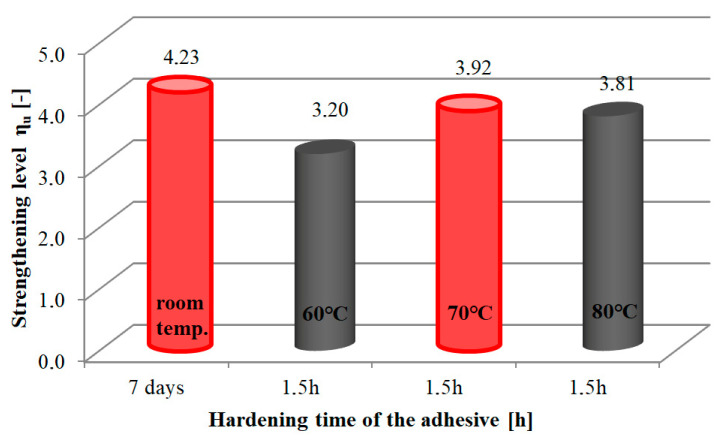
Graphical representation of the level of strengthening of RC elements.

A graphical representation of the strengthening level of RC elements is shown in Figure 6. 

It was found that the accelerated adhesive curing time obtained by heating provides the strengthening level similar to that of the procedure without heating. The strengthening level ranged from 3.20 to 3.92. The highest value of the heated elements was observed at a temperature of 70 °C. This was the closest value to that of the strengthening level (*η_u_* = 4.23) in the procedure without accelerating the cure time (see Figure 6). Compared to the highest strengthening effect of *η_u_* = 3.92, the levels at strip heating temperatures of 80 °C and 60 °C were lower by 2.8% and 18.4%, respectively. 

It was therefore concluded that:

- the use of strip heating in the NSMR method significantly reduces the adhesive hardening process from 7 days to 1.5 h, thereby reducing the duration of the entire component strengthening process;
- the adhesive cure time of 1.5 h and a strip heating temperature of 70 °C are optimal for strengthening RC elements with near surface mounted CFRP strips; the prototype heating device used in the study makes it possible to significantly reduce the adhesive cure time.

## 3. Materials and Method 2

In order to verify the effectiveness of the accelerated process achieved by heating the strip during structural strengthening, tests were performed on RC beams at the predetermined optimal heating temperature and adhesive cure time. The test program for the RC beams also allowed the effectiveness assessment of the strengthening system installed on the underside of NSMR elements in flexure.

### 3.1. Research on the Effectiveness of Strengthening Reinforced Concrete Beams Using the Optimal Temperature and Time of Heating CFRP Strips

#### 3.1.1. Test Stand

**Figure 7:**
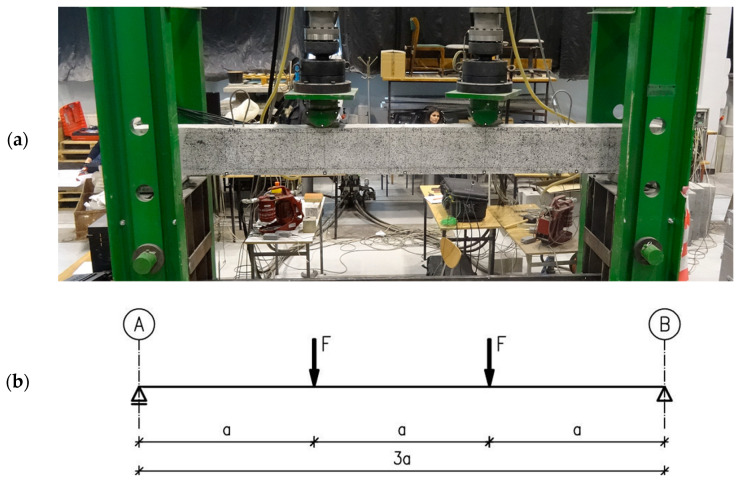
View of the test stand with the component under test (**a**) and static scheme (**b**).

The test stand for testing single-span beams was adjusted for strengthening from the bottom up of the beams under load. The stand is a steel frame with two columns connected by an overhead transom that allows testing beams with a length l_eff_ = 3.00 m. Loads are applied via hydraulic cylinders suspended from the frame’s top transom. Bridge bearings provide support for the beams (sliding and non-sliding). The reinforced concrete beams were tested as simply supported, loaded with two concentrated forces F, at equal distance from the supports, to produce a section of almost constant bending moment. The test stand with the adopted static scheme for testing beams is shown in Figure 7.

To determine load application points, bending failure was assumed for all test specimens. Therefore, the shear rate was calculated, ignoring the dead weight of the beam, according to the formula:

(2) $$D=\frac{M}{Fd}$$

where *M*—the bending moment, *F*—the value of the load applied to the test element, d—the usable height of the section. 

After substituting the bending moment M=F·a into (2) from the adopted static scheme, we get the following formula:

(3) $$D=\frac{M}{Fd}=\frac{Fa}{Fd}=\frac{a}{d},\;a=Dd.\;$$

where *a*—the distance of the applied force from the support.

Assuming that failure at the predominance of the bending moment over the transverse force occurs when the shear rate is between 3.0 and 6.0 [36] and with the assumed d = 257 mm (including the groove for NSMR strengthening), we obtain:

(4) a∈<771mm÷1542mm>

Therefore, the distance of force application point from the supports was assumed to be equal to a = 1000 mm.

A Labtronic measurement set and RS BasTest software as well as a 3D measurement optical system of the ARAMIS type, which allowed the analysis of the damage image, were used to record the load during the test. 

#### 3.1.2. Tested Elements

**Figure 8:**
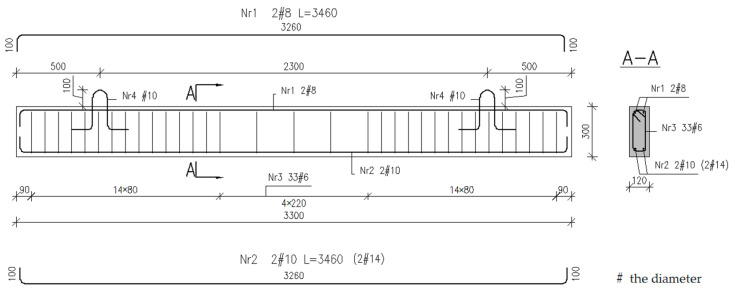
Details of two series of tested beams—steel reinforcement.

In all tested beams with dimensions of 120 × 300 × 3300 mm, ribbed steel bars of A-IIIN grade BST500S were used for longitudinal reinforcement. The elements were divided into two main series I and II, differing in the steel reinforcement ratio. In both series, two 8 mm diameter bars were used in the compression zone. In the tension zone, two 10 mm bars (series I) and two 14 mm bars (series II) were used. The stirrups were made of the same class and grade of steel, with a diameter of 6 mm, spaced at 100 mm (shear section) and 225 mm (pure bending section)—see Figure 8. 

Each series included five beams: one unstrengthened beam (symbol R) and four beams strengthened with single composite strips. The beams were reinforced with two different steel ratios (low: 1 and high: 2) and composite ratios (low W: 1 and high W: 2). For example, A1W1 denotes that the beam was heated (A), had a low steel reinforcement ratio (1), and was strengthened (W) at a low composite ratio (1). Sika^®^CarboDur^®^ S NSM 1.030 and Sika^®^CarboDur^®^ S NSM 2.025 systems were used for strengthening. Different adhesive cure times were used (symbol A –1.5 h, symbol C—7 days). The cure time of 1.5 h was achieved by heating the strip with the prototype heating device. Tests element data are summarised in Table 3.

#### 3.1.3. Loading Program and Strengthening of Elements 

**Figure 9:**
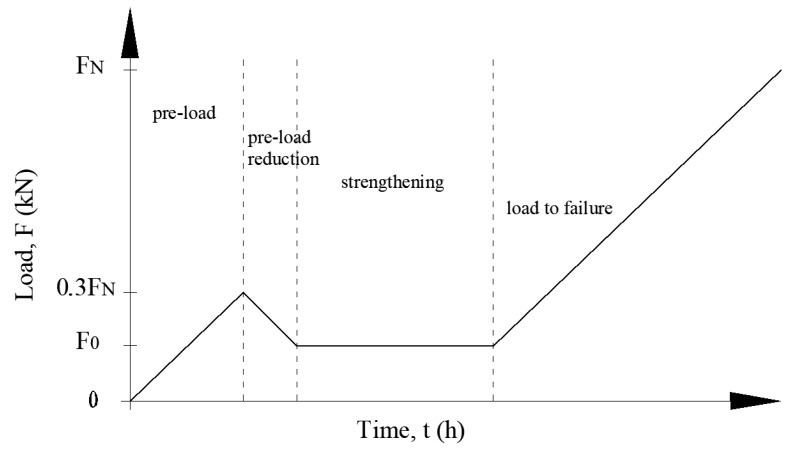
Loading program for beams strengthened with CFRP strips [33].

To simulate the strengthening of RC elements under real conditions, preloaded elements were strengthened on the underside. The load was maintained throughout the adhesive cure time. The assumed preloading corresponded to the actual loads on the RC floor beam. It was thus assumed that the value of the preloading force would increase to 30% of the unreinforced beam load capacity and reach 8 kN and 13 kN for beams with lower and higher steel reinforcement ratios, respectively. Accordingly, the preloading was reduced to 3.9 kN for 2#10 and 6.6 kN for RC beams 2#14—and maintained at the same level throughout the strengthening procedure. The load was maintained both in the accelerated strengthening method (1.5 h) and the conventional method (7 days). The elements were loaded and unloaded at the concentrated force F increase rate of 0.4 kN/min. After the entire strengthening process was completed, the reinforced concrete beams were loaded monotonically to failure. A graphical representation of the loading program is shown in Figure 9.

**Figure 10:**
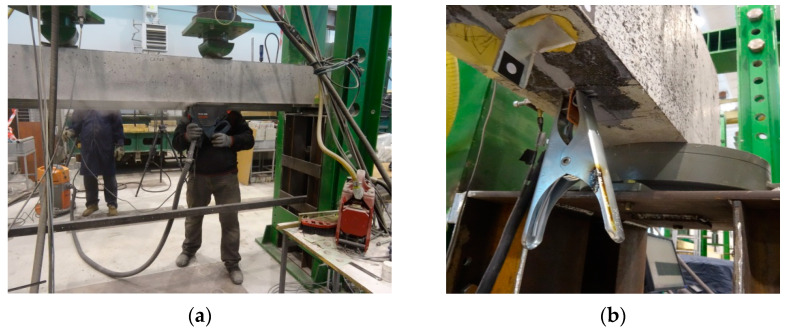
The process of strengthening reinforced concrete beams: (**a**) Making a groove from the bottom surface of the beams; (**b**) View of the copper flat bar with the current wire connected [33].

The strengthening procedure started with the substrate preparation for installing a CFRP strip. The groove in the concrete cover was cut out on the test stand, from the bottom up of the components under the load described above (see Figure 10a). The grooves were made using a diamond blade concrete saw. Their dimensions were taken in accordance with the requirements of the strip and adhesive manufacturer [37] and were 7 × 14 mm and 7 × 24 mm for the Sika^®^CarboDur^®^ S NSM 1.030 and Sika^®^CarboDur^®^ S NSM 2.025 systems, respectively. The groove was made at a distance of 60 mm from the side edge of the tested element. Its length was determined by the “capability” of the concrete saw, i.e., its ability to reach the steel support as far as possible. Before bonding, the groove was thoroughly cleaned of concrete residue and dust using compressed air. The CFRP strip was then cut to the appropriate length and bonded into the concrete cover using the adhesive of the system. As the length of the strip depended on the groove length, the lengths of the Sika^®^CarboDur^®^ S NSM 1.030 and Sika^®^CarboDur^®^ S NSM 2.025 strips were 2360 mm and 2260 mm, respectively. The cleaned groove was then filled with the adhesive and the strip was inserted. The beam was left under a constant load (3.9 kN and 6.6 kN for RC beams 2#10 and 2#14, respectively) for 7 days under laboratory conditions at 23 °C. The beams with the adhesive subjected to accelerated curing (1.5 h) at a heating temperature of t = 70 °C had the ends of the strip smeared with copper paste before bonding. Copper flat bar clips were clamped on the strip ends, extending beyond the tested element (see Figure 10b) to allow the current to flow between the conducting wires of the heating device and the carbon fibre strip. The so-prepared strip was subjected to heating using the prototype heating device (see Figure 2). 

## 4. Results 2

### 4.1. Beam Failure Mechanism

**Figure 11:**
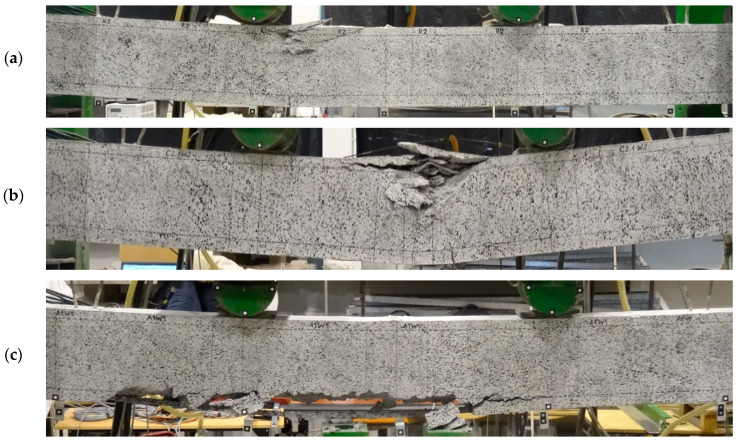

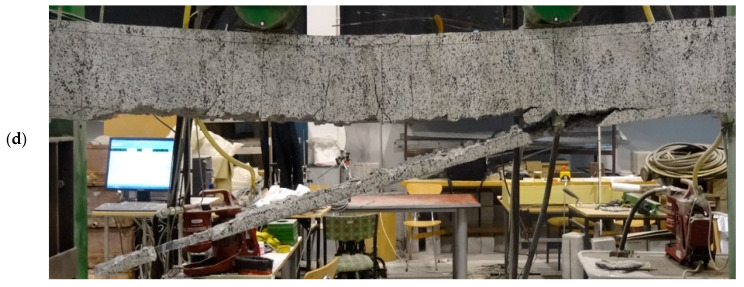
Failure modes of RC beams (examples): (**a**) Non-strengthened beam; (**b**) C2.1W2 beam; (**c**) A1W1 beam; (**d**) C1W2 beam.

Non-strengthened beams (R1 and R2) failed in a manner typical for flexural RC elements by exhausting the load-bearing capacity of the steel and concrete in the compression zone (see Figure 11a). The crushing of the concrete took place in the zone of an almost constant bending moment, near the loading force, located on the side of the hinged-sliding support. Two failure mechanisms were observed in the strengthened beams—debonding of the composite strengthening system along with the concrete cover (see Figure 11c,d) and concrete crushing in the compression zone of the section (see Figure 11b). 

Failure due to composite debonding was observed in four strengthened beams, among which two failure modes were further distinguished: type A and B. Type A was characterized by the fact that within the connection between the strip and the copper flat bar (necessary for power supply) the concrete cover remained well bonded to the strip, and the composite (in this part) did not debond from the reinforced concrete beam under test—beams A1W1, A2W1 (see Figure 11c). The second type of failure mechanism—type B—was characterized by a plane of debonding running along the steel reinforcement, which covered the area and the connection between the strip and the copper flat bar. The flat bar strip and concrete cover debonded from the tested element, resulting in a characteristic “scramble”—A1W2, C1W2 beam (see Figure 11d). Another failure mode of the strengthened beams was exhausting the load-bearing capacity in the compression zone of the concrete—A2W2, C2W1, C2.1W2 beams (see Figure 11b). The test of the C1.1W1 beam was automatically stopped by the program controlling the actuators once the displacement limit set at 70mm was reached.

### 4.2. Strengthening Level of the Beams

The actuator controller and the DIC optical system simultaneously recorded the reported force values. The recorded maximum F*_N_* values are summarized in Table 4. With the adopted force application points at a distance of 1 m from the supports, the magnitude of the bending moment is numerically equal to the force. The moments at which the beams failed were used to determine the strengthening ratio of the tested elements in relation to non-strengthened elements. The strengthening level *η_u_* was calculated using Formula (1) and is shown in Table 4. The steel reinforcement ratio $\rho_{s}$ and the composite ratio $\rho_{f}$ were calculated from:

(5) $$\rho_{s}=\frac{A_{s}}{bd_{s}},\;\rho_{f}=\frac{A_{f}}{bd_{f}}.$$

where *A_s_*_—_area of steel reinforcement; *A_f_*_—_area of composite strip; *b*—section width; *d_s_*—usable height from the beam compression face to the centroid of the steel reinforcement; *d_f_*—usable height from the beam compression face to the centroid of the composite reinforcement.

The strengthening levels of the RC beams ranged from *η_u_* = 0.23 to *η_u_* = 0.95. It can be concluded that the efficiency obtained using the NSMR method without heating the strip was lower than that reported in the literature. It seems that strengthening efficiency of 23% or 38% (beams C2W1, C2.1W2) corresponds to that obtained with externally bonded strips (EBR method) [22,38].

**Figure 12:**
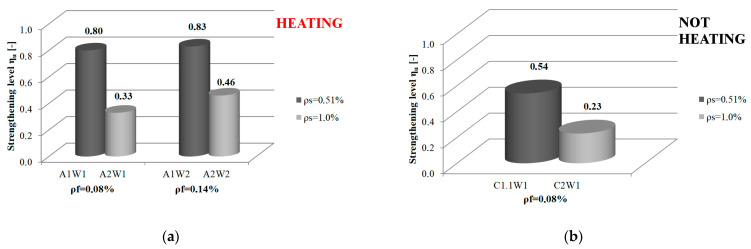
Relationship between strengthening effectiveness and steel reinforcement ratio: (**a**) Beams subjected to an accelerated strengthening process; (**b**) Beams in which the adhesive hardening process took 7 days.

Analysis of the effect of steel reinforcement ratio in NSM soffit-strengthened RC beams under load on the strengthening effectiveness shows and confirms the statement about the lower strengthening level relative to the capacity of non-strengthened elements with higher steel reinforcement ratios (Figure 12). This applies to both values ρ_f_ = 0.08% and ρ_f_ = 0.14%. It was found that the average strengthening efficiency of elements with a steel reinforcement ratio of ρ_s_ = 0.51% was 78% (67% at ρ_f_ = 0.08% and 89% at ρ_f_ = 0.14%), while for elements with a steel reinforcement ratio of ρ_s_ = 1.00%, it was 35% (28% and 42%, respectively). 

The same trend of lower strengthening of beams with a higher steel reinforcement ratio can also be seen for beams undergoing accelerated strengthening (see Figure 12a). The strengthening level of beams with a lower steel reinforcement ratio is higher (*η_u_* = 0.80 for ρ_f_ = 0.08% and *η_u_* = 0.83 for ρ_f_ = 0.14%) than that of beams with a steel reinforcement ratio of ρ_s_ = 1.00%. (*η_u_* = 0.33 and *η_u_* = 0.46, respectively). 

Doubled steel reinforcement ratio of the tested beams (from ρ_s_ = 0.51% to ρ_s_ = 1.00%) provides an almost 60% reduction in the strengthening level, regardless of whether the adhesive cured 7 days or 1.5 h. A lower strengthening level was observed for longer adhesive cure times (a decrease of 57.4% for ρ_f_ = 0.08% and 60% for ρ_f_ = 0.14%). When the accelerated strengthening process was used, the decrease was 58.8% and 46.8%, respectively. Therefore, it can be concluded that using “hot” curing with the system adhesive is beneficial. 

### 4.3. Effect of Heating the Strip—Accelerated Strengthening Process

Comparing the strengthening levels of beams with “heated” and “non-heated” strips in terms of the same steel and composite reinforcement ratios, it can be seen that the accelerated cure process positively affects the strengthening effectiveness. Strengthening efficiency is generally higher for beams with accelerated adhesive curing. For beams with a steel reinforcement ratio ρ_s_ = 0.51% and a low composite ratio, the strengthening level increased by 48.1% (beam A1W1 compared to beam C1.1W1). For beams with a steel reinforcement ratio of ρ_s_ = 1.00%, an increase of 43.5% (beam A2W1 compared to beam C2W1) and 21.1% was achieved (beam A2W2 compared to beam C2.1W2). One beam, C1W2, with low steel and high composite reinforcement ratios, did not confirm the trend. In this case, there was a 14% increase in the strengthening level of the beam without the heated strip (C1W2) compared to the beam with the heated strip (beam A1W2). 

**Figure 13:**
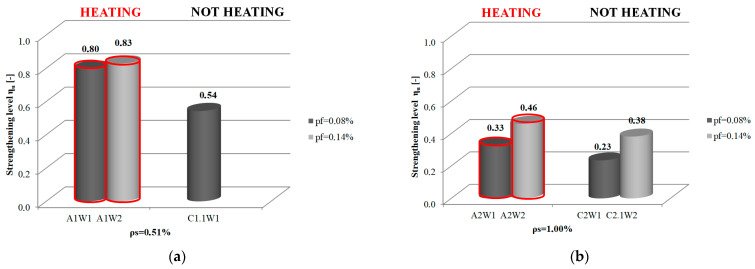
Graphical representation of the strengthening level of RC beams: (**a**) Beams with low steel reinforcement ratios; (**b**) Beams with high steel reinforcement ratios– red colour indicates the beams strengthened with heating of composite strip.

A graphical representation of the strengthening level of beams wit steel reinforcement ratios of ρ_s_ = 0.51% and ρ_s_ = 1.00% and two CFRP reinforcement ratios ρ_f_ = 0.08% and ρ_f_ = 0.14% for monotonic force increments until element failure is shown in Figure 13. The figure also illustrates the difference in the strengthening ratio of beams strengthened using accelerated curing (A-series beams) and strengthened without heating the strip (C-series beams). The C1W2 beam was the only one of the 10 beams tested that failed to show the overall trend observed in the comparison of elements in the “hot” and “cold” methods and steel and composite reinforcement ratios. For this reason, it was disregarded from further analysis.

**Figure 14:**
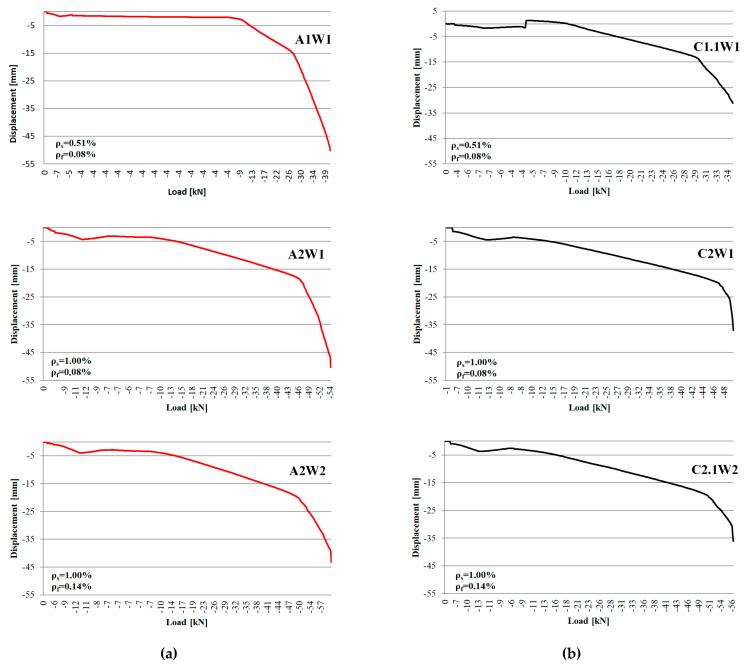
Load vs. displacement in beams during adhesive curing and failure loading, (**a**,**b**)—heated and non-heated beams with the same reinforcement ratios.

Figure 14 shows a graphical representation of the load-vertical displacement relationship in the mid-span of the reinforced beams during adhesive curing and loading to failure. The red line marks the beams that were strengthened with heated strip, while the black line marks the beams where heating was not applied. The comparison of the identically reinforced beams indicates greater deflections at the moment of reaching breaking forces in the beams strengthened with the accelerated procedure. This confirms greater efficiency of the accelerated strengthening method. 

## 5. Discussion

Strengthening the structure of a building is necessary when a deficiency in load-bearing capacity or stiffness in relation to the operational needs is detected. It can be due to long time in service, or other reasons such as changes in service loads. Nowadays, composite materials, which are expensive but have numerous very positive features, are increasingly used for strengthening, and research is being conducted extensively into their effective use. Among other ways to increase the effectiveness of passive strengthening of reinforced concrete elements is the bonding of composite strengthening into the concrete cover, called the NSMR method. 

This paper presents the results of a study of the effectiveness of strengthening with CFRP-type composite materials of reinforced concrete beams using the NSMR method in a way that simulates real the real on-site conditions. 

Tests were performed on beams strengthened in a way that simulates the actual way of strengthening, i.e., from the bottom up of the beams, under loads corresponding to permanent loads. Acceleration of the strengthening time was achieved by reducing the adhesive curing time to 1.5 h by heating the CFRP strip to 70℃, which was determined to be the most effective temperature (see Section 2 and Section 3). 

The results obtained in the study led to the following conclusions:

- Strengthening RC beams under load on soffits in the NSM method increases the failure load compared to non-strengthened beams.

This applies both to the use of a composite ratio of ρ_f_ = 0.08% and ρ_f_ = 0.14%. The increase in failure load applies both to beams with low and high steel reinforcement ratios (ρ_s_ = 0.51%, ρ_s_ = 1.00%, respectively).

- The use of strip heating reduces the adhesive cure time.

With the SikaDur^®^-330 system, the curing time was reduced from 7 days to 1.5 h, which translates directly into reduced execution time for the entire strengthening process. Thus, strip heating during strengthening can help reduce economic and social costs related to taking a part or the entire reinforced structure out of service.

- The strip heating temperature of 70 °C established in the research can be taken as the optimal temperature used to reduce the adhesive cure time.

Compared with the non-strengthened elements, the average strengthening level of the RC elements tested for this purpose was equal to *η_u_* = 3.92 and was most similar to the average strengthening level achieved when the curing time was 7 days (*η_u_* = 4.23).

- A temperature of 70 °C maintained while heating the strips for 1.5 h is sufficient to effectively reduce adhesive cure time.
- Reducing the adhesive cure time by heating the composite strip results in satisfactory strengthening efficiency.

The average value of the strengthening level in relation to non-strengthened elements is in the range *η_u_* = 3.20 ÷ *η_u_* = 3.92.

- Increased breaking load was observed in the case of both methods (accelerated and non-accelerated).

The value of the breaking load for beams with “heated” and “unheated” strips increased from 32.85% to 82.82% and from 23.46% to 54.31%, respectively.

- The efficiency of strengthening RC beams with and without heating composite strips for bonding in the NSM method depends on the steel reinforcement ratio.

The higher the reinforcement ratio, the lower the strengthening efficiency (Figure 12). Doubled steel reinforcement ratio (ρ_s_ = 0.51% to ρ_s_ = 1.00%) results in an almost 60% drop in the strengthening level, regardless of whether the adhesive cure time was 7 days or 1.5 h.

- Strip heating significantly increases the strengthening level compared to that of beams where no heating was used.

In the area of the same steel reinforcement ratio, there is a noticeable increase in the strengthening level (by up to 50% for beams with ρ_s_ = 0.51%). A slightly smaller increase in the strengthening level was found in beams with a steel reinforcement ratio of ρ_s_ = 1.00%, which was 43%. In the case of beams heavily reinforced with steel and composite strips (ρ_s_ = 1.00%, ρ_f_ = 0.14%), the increase in the strengthening level with strip heating compared to the method where heating was not used is slightly smaller, but still noticeable, 21%.

- The use of strip heating during strengthening of RC beams increases the value of deflections at the load level corresponding to the breaking force in relation to the deflections of strengthened beams where heating was not used. This confirms the achievement of greater reinforcement efficiency with the accelerated strengthening method.

The increase in deflection is observed regardless of the steel reinforcement ratio.

## 6. Conclusions

The obtained results are very important in terms of achieving a much higher efficiency of strengthening elements in flexure when using the strip heating technique in the NSMR method. This additionally allows reducing the adhesive cure time and therefore the strengthening execution time.

It seems that the research should be continued, first of all, to clarify the mechanism for the higher strengthening efficiency with the strip heating technique. It also seems important to determine the steel reinforcement ratio (Figure 12) above which this strengthening method should be modified or not used at all.

## Figures and Tables

**Table 1:** Tested specimen data.

Series	Elements	Adhesive Cure Time [h]	Adhesive Cure Temperature [℃]
1 ^1^	A1÷A3	-	-
2	B1÷B3	1.5	60
3	C1÷C3	1.5	70
4	D1÷D3	1.5	80
5	E1÷E3	168.0 (7 days)	-

^1^ Non-strengthened elements.

**Table 2:** Values of destructive forces and the designated strengthening level.

Series	Tested Elements	F_N_ [kN] ^1^	M_N_ [kNm] ^2^	M_Nśr_ [kNm] ^3^	*η_u_*
1	A1	10.9	1.36	1.40	-
	A2	11.5	1.44		
	A3	11.3	1.41		
2	B1	44.6	5.58	5.90	3.20
	B2	48.8	6.10		
	B3	48.1	6.01		
3	C1	54.9	6.86	6.90	3.92
	C2	56.9	7.11		
	C3	53.9	6.74		
4	D1	51.8	6.48	6.75	3.81
	D2	56.8	7.10		
	D3	53.5	6.69		
5	E1	59.5	7.44	7.35	4.23
	E2	57.5	7.19		
	E3	59.3	7.41		

^1^ F_N_—destructive force; ^2^ M_N_—destructive moment; ^3^ M_Nśr_—destructive moment, mean value.

**Table 3:** Test elements.

Series	Beams	Rebar in Compression/Tension	CFRP Strip n × tf × bf [mm]	Adhesive Cure Time [h]	Adhesive Cure Temperature [℃]
1	R1	2#8/2#10 ^1^	-	-	-
	A1W1		1 × 10 × 3.0	1.5	70
	A1W2		1 × 20 × 2.5	1.5	70
	C1.1W1		1 × 10 × 3.0	168.0 (7 days)	23
	C1W2		1 × 20 × 2.5	168.0 (7 days)	23
2	R2	2#8/2#14 ^1^	-	-	-
	A2W1		1 × 10 × 3.0	1.5	70
	A2W2		1 × 20 × 2.5	1.5	70
	C2W1		1 × 10 × 3.0	168.0 (7 days)	23
	C2.1W2		1 × 20 × 2.5	168.0 (7 days)	23

^1^ #—diameter.

**Table 4:** Breaking moment and composite reinforcement ratios of the beams.

Series	Elements	ρ_s_ [%]	ρ_f_ [%]	F_N_ [kN]	M_N_ [kNm]	*η_u_*
1	R1	0.51	-	22.61	22.61	-
	A1W1		0.08	40.65	40.65	0.80
	A1W2		0.14	41.34	41.34	0.83
	C1.1W1		0.08	34.89	34.89	0.54
	C1W2		0.14	44.02	44.02	0.95
2	R2	1.00	-	41.06	41.06	-
	A2W1		0.08	54.54	54.54	0.33
	A2W2		0.14	59.97	59.97	0.46
	C2W1		0.08	50.69	50.69	0.23
	C2.1W2		0.14	56.72	56.72	0.38

## Data Availability

The data presented in this study are available upon request from the corresponding author.
